# Smartphone-based behavioral monitoring and patient-reported outcomes in adults with rheumatic and musculoskeletal disease

**DOI:** 10.1186/s12891-022-05520-5

**Published:** 2022-06-11

**Authors:** Elizabeth Mollard, Sofia Pedro, Rebecca Schumacher, Kaleb Michaud

**Affiliations:** 1 University of Nebraska Medical Center, Omaha, NE USA; 2 FORWARD, The National Databank for Rheumatic Diseases, Wichita, KS USA

**Keywords:** Rheumatic and musculoskeletal disease, Mobile health, Patient reported outcomes, Physical function

## Abstract

**Background:**

Rheumatic and musculoskeletal diseases (RMD) are associated with depression, fatigue, and disturbed sleep – symptoms that often impact behavior and activity. Patient reported outcomes (PROs) are a way of collecting information on the patient symptom experience directly from the individual. The purpose of this study was to measure and compare user smartphone sensor and activity data in adults with RMDs and assess associations with PROs.

**Methods:**

We invited adults with RMDs enrolled in the FORWARD Databank to participate by installing a custom app on their smartphone and answering PROs (pain, global, HAQ-II) questions daily and weekly over 3 years. Passive data collected included mobility distance, unique calls and text messages, call durations, and number of missed calls. Confounders included sociodemographic, clinical, passive phone behavior, and seasonal factors. Kappa statistics between PRO and flares were computed to measure agreement. The agreement between daily and weekly VAS pain was estimated using the intraclass (ICC) correlation of a two-way random effect model. The relationship between the weekly PRO outcomes and the passive phone data was analyzed with a linear mixed-effect model (LMM), including a random intercept for participant and slope for time in the study with an unstructured covariate structure.

**Results:**

Of the 446 participants, the mean (SD) age was 54 (12) years, most (65.5%) had rheumatoid arthritis (RA), the vast majority (91%) were female, and the US Northeast has the least representation (12%). Longer reaction times, interaction diversity, and higher mobility were associated with worse PROs while longer text messages were associated with better PROs. Participants in this study showed good levels of adherence which holds promise for future interventions using passive behavior measures in self-management and clinical follow-up.

**Conclusion:**

This is the first study to examine passive smartphone behavior with PROs in RMDs and we found significant associations between these behaviors and important health outcomes of pain and function. As smartphone usage continues to change, future studies should validate and expand on our findings with a goal of finding changes in patient symptoms passively through mobile device monitoring.

## Background

Mobile smartphones have become a standard tool for healthcare delivery and health self-monitoring [1]. Smartphones are equipped with various sensors which can measure movement, activity, sleep, and geographical location. Sensor data combined with user data such as which applications are used, for how long, and in what capacity can give information about an individual’s activity, behavior, and personality [2, 3]. Combining this passively collected data (sensing data and user activity data) allows scientists to make assumptions about behavioral patterns [4].

Patient-reported outcomes (PROs) are traditionally considered health outcomes as reported by the individual patient [5]. Whereas the clinician is limited to measurement of health variables within the healthcare setting, PROs are valuable because they measure the individual’s experience and capture a “real-time” ongoing clinical picture of their quality of life, symptomatology, and disease experience [5, 6]. PROs are essential for understanding the day-to-day experience of living with an illness and may improve patient communication, decision making, satisfaction, and confidence [7].

Using passively collected data from a smartphone to collect information about the individual in their day-to-day life expands upon the principle of PROs while also reducing the bias of self-report [6]. Collecting PROs combined with passive smartphone data is a novel way of incorporating the individual symptom experience with objective behavioral data outside of the clinical setting. Additionally, combining PRO and passive data allows scientists to better draw generalizations about behavioral patterns and health status. While this data combination has been utilized to predict disease course in psychiatric populations, limited information is available on individuals with chronic disease [8].

Individuals with rheumatic and musculoskeletal diseases (RMD) like rheumatoid arthritis (RA) have daily fluctuations in associated symptoms, including pain, fatigue, physical disability, and mood disturbances [9]. These symptoms are associated with alterations in activity. As symptoms increase in frequency or severity, physical activity levels and cognitive response times tend to decrease [10]. When individuals are well, and symptoms subside, activity levels and cognitive processing times increase. These symptom and disease burden associated activity changes have the potential to be measured through smartphone sensing technology and have been correlated to rheumatic illness activity [11].

Smartphones collect passive data on individuals without requiring adherence to survey tool completion, which is a common PRO challenge. Barriers to PRO include the time required to complete the PRO tools, difficulty using electronic devices to complete PROs, and that an individual is less likely, or unable to complete PRO tools when they are unwell [12, 13]. Passive smartphone activity data collected throughout the day and over time has the potential to predict individual health changes like disease flare. Combining smartphone user activity data with PROs would provide more in-depth real-time data about the individual’s clinical picture.

Several studies exist that focus on wearable activity trackers and their relationship to RMD disease activity, however, there is limited literature available on how passive smartphone user data may correlate with RMD PROs. Available literature suggests that research on RA symptoms and smartphone passive data has been found to be feasible, with high levels of patient engagement and may predict RA disease activity [14]. Additionally, sensor measures such as wrist motion which may quantify meaningful RMD specific symptoms have also been shown to be feasible [15]. These findings demonstrate the potential to quantify meaningful RMD clinical information passively and remotely from an individual’s smartphone.

Using passive smartphone data combined with PROs may improve symptom monitoring and management and may have utility as a diagnostic and disease management tool in individuals with suspected rheumatic illness. The purpose of this study was to: 1. Measure and compare user phone activity via smartphone sensing activity in individuals with RMDs (RA and non-RA), and 2. Assess associations between passively collected behavioral data and participant’s PRO disease severity.

## Methods

### Study population

Six hundred and twenty-nine active participants of FORWARD, The National Databank for Rheumatic Diseases, were invited to participate in this study based on their prior response of owning a smartphone and expressing willingness to be invited. The FORWARD Databank is an ongoing United States (US)-based longitudinal observational study that enrolls participants from rheumatology clinics [16]. Participants complete semi-annual, comprehensive questionnaires covering several aspects of their diseases, including demographics, clinical characteristics, disease severity, and treatments.

To be eligible to participate, individuals were required to: (1) be over 18 years old (2); own a smartphone with an iOS (iPhone) or Android operating system (3); be willing to download and use an app to respond to daily or weekly surveys and share passive measures on smartphone activity (4); have an RMD diagnosis including rheumatoid arthritis (RA), systemic lupus erythematosus (SLE), osteoarthritis (OA), fibromyalgia, and musculoskeletal chronic pain.

### Data sources

Two data sources were used in the study, including the custom created smartphone application (hereby called “the App”) data collected via smartphone (passive phone data and PROs) and the FORWARD questionnaires (enrolment and 6-month phase), which were linked using the most immediate prior FORWARD questionnaire from the day participants started using the app.

### The app data

The PROs that were selected were chosen due to their common use in rheumatology populations in both research and clinical practice, and our experience using these specific measures in our research [16]. No additional measures were selected to limit subject burden. The PROs that were collected included the daily Visual Analogue Scale (VAS) pain and physical function and weekly VAS Pain, VAS global patient assessment, and HAQ-II; these last three variables combined mean formed the Patient Activity Scale-II (PAS-II), a measure of RMD activity. Daily passive measures describing smartphone activity (collected in Android only) included the number of unreturned calls, average duration per call (minutes), the average length of SMS per message (characters), call count, SMS text message count, interaction diversity (number of unique phone numbers), missed interaction (including texts and calls) and aggregated communication (number of calls and SMS). Mobility as the approximate distance (in miles) covered while walking, biking, running, etc. was collected with the intent to capture physical activity; mobility radius (in miles), an approximate for overall traveling, measured the radius of the circle around the locations gathered for the participant that day. Additionally, the time-of-day participants completed their PROs on their phone (morning, afternoon, and evening/night), season (winter, spring, summer, and fall), working days versus weekends, and reaction time (in hours, the length of time taken by a participant to respond to a given questionnaire). Passively collected measures were limited to when participants kept their smartphone active and with them.

### FORWARD data

FORWARD variables included demographics, such as diagnosis (RA vs. non-RA), age, sex, race, educational years, employment status, marital status, total household income (US dollars thousands), insurance (Medicare vs. other), number of persons living in the household; clinical and severity variables such as Rheumatic Disease Comorbidity Index (RDCI), Health Assessment Questionnaire-II (HAQ-II), Patient Activity Scale-II (PAS-II), Rheumatoid Arthritis Disease Activity Index (RADAI), EuroQoL-5D (EQ-5D), SF-36 Physical Component Summary (PCS) and Mental Component Summary (MCS) scores, and Visual Analog Scales (VAS), which included pain, fatigue, and patient global assessment. Geographical area (rural versus urban) and US region defined by first zip code digit (Southeast, Northeast, Midwest, West) were also considered to possibly affect smartphone behavior and health outcomes.

### Procedures

This study was conducted in two phases that took place over 3 years. During Phase I (Sep 2013 to May 2014), 190 participants downloaded the app and agreed to respond to daily VAS pain and HAQ-II for 3 months and weekly to VAS pain, global assessment, and HAQ-II. Participants also agreed to share phone activity and sensing data, including the number of unique calls and texts, duration of calls, distance traveled, etc.

For Phase II (May 2013 to August 2015), 256 new subjects enrolled. Identical in data collection, this phase included an update on flare assessment and improved graphics. Phase I participants who were willing to continue were allowed to participate in the subsequent phase.

### Statistical analysis

Baseline characteristics and phone/passive measures were compared by diagnosis using T-tests and Chi-square/Fisher’s test for continuous and categorical variables, respectively, i.e., when participants started using the App. Similar analyses were performed comparing phone operating systems as iOS and Android had different rules on what data from the smartphone would be made available to the App. Correct adherence to the App was computed daily and weekly over the study period to the point of discontinuation (i.e., no App use ≥ 1 month (implementation)). Kaplan Meier estimates were used to analyze time to discontinuation and Cox regression for predictors. Confounders included sociodemographic, clinical, passive phone behavior, and seasonal factors. Kappa statistics between PRO and flares were computed to measure agreement.

The agreement between daily and weekly VAS pain was estimated using the intraclass (ICC) correlation of a two-way random effect model, with the method as the first level (daily or weekly pain) and the participant as the second level. If the agreement between daily or weekly measures is high, the weekly measurement was preferred, imputing each weekly value in the corresponding seven prior days. The agreement analysis was limited to the first 3 months, where both weekly and daily measures were collected.

Passive mobile variables were log-transformed due to the skewness of the distributions and further transformed using moving averages of the previous 7 days (sensitivity analyses with different lags). The relationship between the four weekly PRO outcomes (pain, global assessment, HAQ-II, and PAS-II) and the passive phone data was analyzed with a linear mixed-effect model (MRM), including a random intercept for participant and slope for time in the study with an unstructured covariate structure. Random intercept models were also compared with random slope models using likelihood ratio tests.

Best models (for fixed effects) were obtained using backward selection with a 20% significance level at which variables could be removed from the models, estimated in the two samples: overall sample and for RA participants only. The saturated model started with the following list of covariates (as fixed effects) for the overall sample: diagnoses, sex, age, white ethnicity, total income, education level, number of persons living in the household, RD comorbidity index, education years, employment status, prior HAQ-II, prior pain, sleep scale, phase initiators, study phase, season, region and interaction between season and region, rural area, workdays, time of day, time in the study as a quadratic function and moving averages of passive data: mobility, mobility radius, average SMS length, average call duration, interaction diversity, missed interactions and log of reaction time. Disease-modifying antirheumatic drugs (DMARDs) or biologic treatment were also included for RA only. All analyses were performed using STATA/MP version 14.2, and all tests were two-sided, with a 5% significance level.

## Results

### Sample and baseline characteristics

**Fig. 1:**
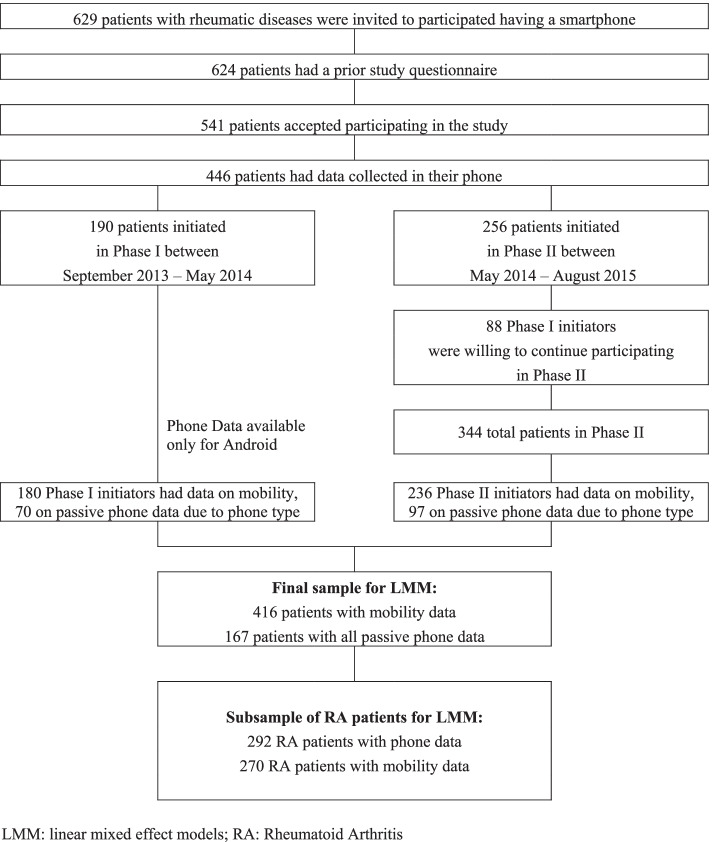
Participant recruitment. LMM: linear mixed effect models; RA: Rheumatoid Arthritis

Of the 624 FORWARD participants with RMDs that were invited to participate, 541 participants accepted, and 446 (292 with RA) downloaded, installed, and had data collected on the study App (Fig. 1). Prior FORWARD questionnaires were collected 2 months (median, IQR 1–3 months) before the start of the study. By phase, the sample split was 190 in Phase I and 256 in Phase II, with 88 participating in both phases. Restricting the sample to Android users having all the passive variables collected, the final sample was composed of 167 participants, 107 with RA diagnosis, with a total of ~ 85,000 time points and 38,000 observations of passive data. The average time follow-up was 60 days (SD 37) for those getting the daily questions in the first 3 months. The average time follow-up for the overall study where weekly questions were asked was 27 weeks (SD 4w) (median 19 weeks).

At initiation, participants were primarily female (91.8%), white (89%), average age of 54 years old (SD 12) with 15 years of education and a mean HAQ-II of 0.78 (SD 0.58) (Table 1). Most participants lived in urban areas (85%) and in midwest-southeast regions (73%). Some differences in disease severity were found between diagnoses such as in PAS-II. Between phone operating systems, iPhone users tended to have better disease severity outcomes and higher total income.

**Table 1:** Baseline characteristics prior to smartphone initiation by diagnosis

Variable – mean (SD) or %	Overall (***N*** = 446)	RA (***N*** = 292)	Non-RA (***N*** = 154)	***P***-value
Age (years)	53.7 (12.2)	54.0 (12.0)	53.0 (12.7)	0.413
Sex (% male)	9.2	9.6	8.5	0.705
Non-Hispanic White (%)	89.77	90.4	88.6	0.558
Married (%)	74.5	73.8	75.8	0.650
Employed (%)	53.1	53.9	51.6	0.655
Education (years)	15.4 (2.2)	15.5 (1.9)	15.2 (2.6)	0.207
Household income (US$1000)	81.5 (40.9)	83.5 (41.0)	77.7 (40.6)	0.160
Number of persons living in household	2.3 (1.1)	2.3 (1.0)	2.4 (1.1)	0.594
Medicare insurance (%)	29.4	29.5	29.2	0.738
HAQ-II (0–3)	0.78 (0.58)	0.75 (0.59)	0.82 (0.56)	0.242
EQ-5D- values	0.75 (0.13)	0.76 (0.13)	0.73 (0.13)	0.037
Physical component score (SF-36)	38.5 (10.7)	39.5 (10.8)	36.7 (10.4)	0.012
Mental component score (SF-36)	48.3 (11.4)	48.7 (11.7)	47.6 (10.8)	0.386
Patient activity score-II (0–10)	3.33 (2.10)	3.11 (2.11)	3.68 (2.02)	0.005
RD comorbidity index (0–9)	2.25 (1.70)	2.14 (1.69)	2.44 (1.72)	0.079
Sleep disturbance (0–10)	4.22 (3.02)	3.93 (3.00)	4.76 (3.00)	0.008
RADAI joint count	7.47 (4.89)	7.69 (5.02)	7.05 (4.63)	0.187
Rural area (%)	14.94	16.32	12.24	0.260
US Region by ZIP code				0.268
Southeast (%)	32.51	35.27	27.27	
Northeast (%)	12.78	11.64	14.94	
Midwest (%)	36.32	34.25	40.26	
West (%)	18.39	18.84	17.53	
Treatment				
csDMARD use (%)		73.88		
bDMARD use (%)		54.64		

*SD* Standard Deviation; *RD* Rheumatic Disease; *HAQ-II* Health Assessment Questionnaire II; *EQ-5D* EuroQoL-5D; *SF-36* 36 item Short Form Health Survey; *RADAI* Rheumatoid Arthritis Disease Activity Index; *csDMARD* Conventional synthetic disease modifying antirheumatic drug; *bDMARD* Biologic disease modifying antirheumatic drug

### Adherence

Overall, 62% of participants reported daily measures in the App (Table 2). Daily and weekly correct implementation was 68 and 90%. The probability of remaining in the study at 6 months was 0.78 and 1 year, 0.64. No differences were found by diagnosis (P log ranks 0.92).

**Table 2:** Baseline characterization of the passive mobile data collected in the App

Mean (SD) or %	Overall (***N*** = 446)	RA (***N*** = 292)	Non-RA (***N*** = 154)	***P***-value (RA vs non- RA)*
*PRO outcomes*				
Daily pain	4.03 (2.33)	3.75 (2.36)	4.57 (2.18)	0.000
Daily function	2.60 (1.78)	2.51 (1.83)	2.78 (1.68)	0.130
Weekly pain	4.16 (2.46)	3.88 (2.41)	4.69 (2.47)	0.001
Weekly global	3.67 (2.36)	3.43 (2.40)	4.11 (2.23)	0.005
Weekly HAQ-II	0.81 (0.55)	0.78 (0.56)	0.88 (0.52)	0.078
Weekly PAS-II	2.88 (1.64)	2.69 (1.65)	3.23 (1.57)	0.002
*Passive data*	*N* = 164	*N* = 109	*N* = 55	
Unreturned calls	1.47 (1.01)	0.53 (1.13)	0.56 (0.86)	
Average call duration/call (minutes)	4.07 (6.76)	4.70 (7.90)	2.81 (3.29)	
Average SMS length per SMS	72.01 (60.01)	70.67 (52.55)	74.66 (72.67)	
Call count	3.98 (4.00)	3.17 (4.16)	3.29 (4.43)	
SMS count	17.45 (23.71)	14.98 (22.24)	22.24 (27.35)	
Interaction diversity	4.52 (4.19)	4.08 (3.83)	4.53 (5.26)	
Missed interactions	0.62 (1.13)	0.68 (1.40)	0.69 (1.12)	
Aggregated communication	19.62 (25.65)	18.17 (23.69)	20.04 (29.88)	
	*N* = 412	*N* = 273	*N* = 143	
Mobility radius	18.79 (96.86)	17.53 (85.09)	11.10 (35.31)	
Mobility	1.52 (1.66)	1.44 (1.73)	1.34 (1.66)	
*Passive data, natural log*				
Unreturned calls	0.25 (0.46)	0.28 (0.49)	0.193 (0.39)	0.215
Average call duration/call (minutes)	0.67 (1.19)	0.76 (1.24)	0.50 (1.07)	0.189
Average SMS length per SMS	4.06 (0.67)	4.03 (0.7)	4.11 (0.57)	0.470
Call count	0.98 (0.87)	1.0 (0.9)	1.0 (0.8)	0.74
SMS count	2.11 (1.30)	2.1 (1.3)	2.1 (1.2)	0.99
Interaction diversity	1.21 (0.77)	1.20 (0.75)	1.22 (0.82)	0.872
Missed interactions	0.32 (0.51)	0.34 (0.52)	0.29 (0.48)	0.552
Aggregated communication	2.27 (1.27)	2.24 (1.28)	2.32 (1.26)	0.726
Mobility radius	1.20 (2.01)	1.25 (2.07)	1.11 (1.89)	0.488
Mobility	−23 (1.43)	−0.21 (1.44)	−0.26 (1.41)	0.716
Time of day of measurements	*N* = 446	*N* = 292	*N* = 154	
morning (%)	28.14	28.08	28.26	0.140
afternoon (%)	45.98	43.08	51.45	
evening/night (%)	25.88	28.85	20.29	
Reaction time (in hours)	2.11 (0.28)	2.13 (3.83)	2.08 (5.13)	0.937
Working days (%)	76.91	75.34	78.87	0.281
Season				0.512
winter (%)	47.75	45.17	52.60	
spring (%)	9.01	9.31	8.44	
summer (%)	10.59	11.38	9.09	
fall (%)	32.66	34.14	29.87	

*SD* Standard Deviation; *RA* Rheumatoid Arthritis; *RD* Rheumatic Disease; *HAQ-II* Health Assessment Questionnaire II; *PAS-II* Patient Activity Scale-II; *SMS* Short Message Service

Younger age HR 0.98 (95% CI 0.97–0.99), pain 1.08 (1.02–1.15), HAQ-II 0.75 (0.60–0.94), Global 1.06 (1.00–1.13), PAS-2 1.13 (1.02–1.24), and summer vs other seasons 2.98 (1.94–4.56) were significant predictors of discontinuation using univariate cox regression.

### Phone data

**Fig. 2:**
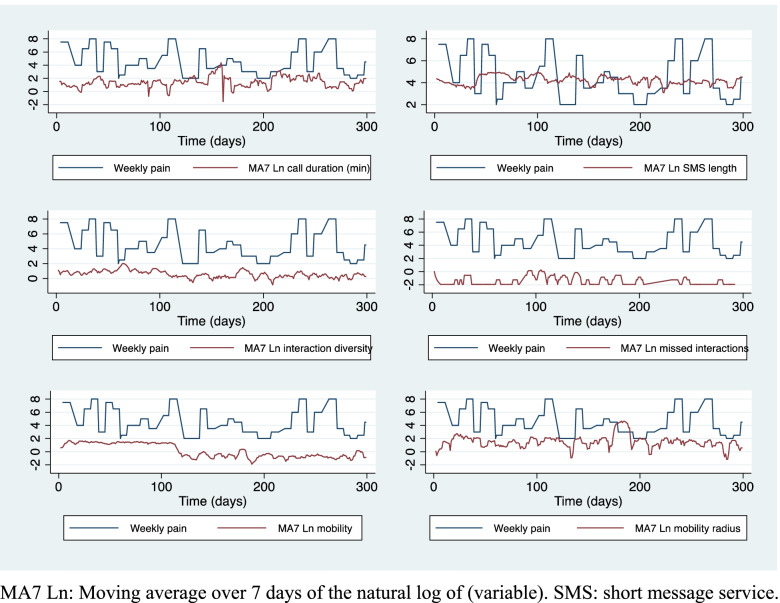
Example of a mobile data phone and weekly-imputed pain of a random participant. MA7 Ln: Moving average over 7 days of the natural log of (variable). SMS: short message service

On average, participants had four calls per day with a duration of 4 minutes per call, sent 17 text messages with 72 characters length, interacted with 4 to 5 distinct people and missed one call per day maximum (Table 2). Figure 2 presents an example of the passive phone data for one participant. Passive data variables were log-transformed due to the skewness of the distributions. No differences were found in phone behavior at baseline between RA and non-RA participants, except on some active PROs such as pain, global assessment and PAS-II. Non-RA participants had worse pain, global assessment and disease activity in comparison to RA participants (driven mainly by Fibromyalgia participants), but no differences were found on function or HAQ-II. Participants took 2 hours on average to respond to the question in the app. Most of the measures on the app were collected early in the morning, during the week (77%) and during the winter (48%).

### Agreement between daily and week PROs

The agreement between daily and weekly pain was 83.3%. Based on this estimate, weekly measurements were used during the entire study, imputing the weekly PROs in the previous 6 days of the week.

### The relationship between PROs and passive phone data

The relationship between passive phone data and the PROs outcomes are listed in Table 3 for the overall sample and Table 4 for RA only. Using the predicted values from the MRM models by increasing values of SMS length, interaction diversity, reaction time and mobility variables, keeping all the other variables at mean values. The best final models searched by backward selection are presented in Table 5.

**Table 3:** Estimates for the multivariate model for passive phone data, keeping all the variables at mean values for the overall sample (all diagnoses)

OVERALL				
Passive variable	Pain	Global	HAQ-II	PAS-II
SMS length				
20 characters	3.95 (3.47–4.43)		0.99 (0.87–1.10)	2.92 (2.59–3.26)
50	3.88 (3.41–4.35)		0.97 (0.86–1.08)	2.87 (2.54–3.20)
100	3.82 (3.35–4.29)		0.96 (0.84–1.07)	2.83 (2.50–3.16)
150	3.78 (3.31–4.26)		0.95 (0.84–1.06)	2.80 (2.47–3.14)
500	3.69 (3.19–4.20)		0.93 (0.81–1.05)	2.74 (2.39–3.09)
Interaction diversity				
0	1.81 (0.60–3.02)	2.30 (1.07–3.53)	0.66 (0.42–0.91)	1.62 (0.86–2.39)
1 person	3.65 (3.17–4.1)	3.58 (3.12–4.05)	0.94 (0.82–1.05)	2.73 (2.40–3.07)
3	3.82 (3.35–4.29)	3.70 (3.25–4.15)	0.96 (0.85–1.07)	2.83 (2.51–3.16)
5	3.90 (3.43–4.37)	3.75 (3.30–4.21)	0.97 (0.86–1.08)	2.88 (2.55–3.21)
15	4.07 (3.58–4.55)	3.87 (3.40–4.34)	1.00 (0.88–1.11)	2.98 (2.65–3.32)
30	4.17 (3.67–4.67)	3.94 (3.46–4.43)	1.01 (0.89–1.13)	3.05 (2.70–3.39)
Reaction time				
0.5 hour	3.85 (3.38–4.32)	3.72 (3.27–4.17)	0.96 (0.85–1.08)	2.85 (2.52–3.18)
2.5 h	3.87 (3.40–4.34)	3.73 (3.28–4.18)	0.97 (0.85–1.08)	2.86 (2.54–3.19)
7 h	3.88 (3.41–4.35)	3.74 (3.29–4.19)	0.97 (0.86–1.08)	2.88 (2.55–3.20)
12 h	3.89 (3.42–4.36)	3.75 (3.29–4.20)	0.97 (0.86–1.08)	2.88 (2.56–3.21)
Mobility				
0.5 miles	3.84 (3.37–4.31)	3.70 (3.25–4.15)	0.96 (0.85–1.07)	2.85 (2.52–3.18)
5 m	3.91 (3.44–4.39)	3.80 (3.35–4.26)	0.99 (0.87–1.10)	2.89 (2.56–3.22)
10 m	3.94 (3.46–4.41)	3.83 (3.38–4.29)	0.99 (0.88–1.11)	2.90 (2.57–3.23)
20 m	3.96 (3.48–4.44)	3.86 (3.40–4.33)	1.00 (0.89–1.12)	2.91 (2.58–3.24)
Mobility radius				
1 mile			0.98 (0.86–1.09)	
5 m			0.97 (0.85–1.08)	
10 m			0.96 (0.85–1.08)	
50 m			0.96 (0.84–1.07)	
250 m			0.95 (0.83–1.06)	

*SMS* Short message service; *HAQ-II* Health Assessment Questionnaire II; *PAS-II* Physical Activity Scale II

**Table 4:** Estimates for the multivariate model for passive phone data, keeping all the variables at mean values for RA participants

Passive variable	Pain	Global	HAQ-II	PAS-II
SMS length				
20 characters	3.46 (2.86–4.07)	3.50 (2.98–4.02)	0.98 (0.82–1.13)	2.61 (2.18–3.04)
50	3.32 (2.73–3.91)	3.38 (2.88–3.88)	0.96 (0.80–1.11)	2.51 (2.09–2.93)
100	3.21 (2.62–3.81)	3.29 (2.78–3.79)	0.94 (0.79–1.10)	2.43 (2.01–2.85)
150	3.14 (2.54–3.74)	3.23 (2.71–3.74)	0.93 (0.78–1.09)	2.38 (1.95–2-81)
500	2.96 (2.33–3.60)	3.08 (2.52–3.63)	0.91 (0.75–1.07)	2.26 (1.81–2.70)
Interaction diversity				
0	1.88 (0.40–3.37)	2.40 (0.92–3.88)	0.69 (0.39–0.99)	1.46 (0.51–2.41)
1 person	3.14 (2.54–3.75)	3.25 (2.74–3.77)	0.93 (0.77–1.08)	2.38 (1.95–2.81)
3	3.26 (2.67–3.85)	3.33 (2.83–3.84)	0.95 (0.79–1.10)	2.46 (2.04–2.89)
5	3.31 (2.72–3.91)	3.37 (2.87–3.87)	0.96 (0.80–1.11)	2.50 (2.08–2.93)
15	3.43 (2.82–4.04)	3.45 (2.92–3.97)	0.98 (0.82–1.14)	2.59 (2.16–3.02)
30	3.50 (2.87–4.13)	3.49 (2.95–4.95)	0.99 (0.83–1.15)	2.64 (2.20–3.08)
Reaction time				
0.5 hour	3.27 (2.67–3.86)	3.34 (2.84–3.84)		2.47 (2.05–2.89)
2.5 h	3.30 (2.71–3.89)	3.36 (2.86–3.86)		2.49 (2.07–2.92)
7 h	3.32 (2.73–3.92)	3.37 (2.87–3.88)		2.51 (2.09–2.93)
12 h	3.33 (2.74–3.93)	3.38 (2.88–3.88)		2.52 (2.10–2.94)
Mobility				
0.5 miles		3.31 (2.81–3.81)	0.95 (0.79–1.10)	2.47 (2.04–2.89)
5 m		3.47 (2.96–3.97)	0.97 (0.82–1.13)	2.53 (2.11–2.95)
10 m		3.51 (3.00–4.02)	0.98 (0.83–1.14)	2.55 (2.12–2.97)
20 m		3.56 (3.04–4.08)	0.99 (0.84–1.15)	2.57 (2.14–3.00)
Mobility radius				
1 mile		3.31 (2.80–3.81)	0.96 (0.81–1.12)	
5 m		3.34 (2.84–3.84)	0.96 (0.80–1.11)	
10 m		3.36 (2.85–3.86)	0.95 (0.80–1.11)	
50 m		3.39 (2.88–3.89)	0.95 (0.79–1.10)	
250 m		3.42 (2.91–3.93)	0.94 (0.78–1.09)	

*RA* Rheumatoid Arthritis; *SMS* Short message service; *HAQ-II* Health Assessment Questionnaire II; *PAS-II* Physical Activity Scale II

**Table 5:** Multivariate linear mixed models between weekly PRO and passive data in the overall sample

Weekly:	Pain	Global	HAQ-II	PAS-II
Age (yrs)	0.03 (− 0.01–0.07)			
Income (USD)	0.00* (0.00–0.00)		− 0.00 (− 0.00–0.00)	(0.00–0.00)
Educational level (yrs)	−0.14 (− 0.33–0.05)			
N. of persons in household	− 0.34* (− 0.58 - -0.10)			− 0.18* (− 0.34 - -0.03)
RD Coindex		− 0.05 (− 0.13–0.02)	− 0.02* (− 0.03 - -0.00)	− 0.04 (− 0.09–0.01)
Male Sex	1.38 (− 0.02–2.77)	1.44* (0.16–2.72)	0.38* (0.04–0.72)	1.18* (0.21–2.16)
White			−0.30 (− 0.66–0.07)	
Married	−0.90 (− 1.93–0.13)	−0.78 (− 1.67–0.12)	−0.08	− 0.51 (− 1.21–0.20)
Employed			(− 0.16–0.00)	
Medicare				0.38* (0.04–0.71)
Initiation (2- second cohort; 1- first cohort)	− 0.60 (− 1.48–0.27)		− 0.16 (− 0.38–0.06)	
Phase (II vs I)	−0.40* (− 0.69 - -0.12)	−0.50* (− 0.77–0.22)	−0.02 (− 0.08–0.03)	−0.33* (− 0.51 - -0.15)
MA7 Ln (Mobility)	0.03 (− 0.00–0.06)	0.04* (0.01–0.08)	0.01* (0.01–0.02)	0.02 (− 0.00–0.04)
MA7 Ln (Mobility radius)			−0.00* (− 0.01 - -0.00)	
MA7 Ln (SMS length /SMS)	−0.08 (− 0.17–0.01)		−0.02* (− 0.03 - -0.00)	−0.06* (− 0.11 - -0.00)
MA7 Ln (Call duration min /call)	− 0.04 (− 0.08–0.00)			
MA7 Ln (Interaction diversity)	0.16* (0.07–0.24)	0.11* (0.02–0.20)	0.02* (0.01–0.04)	0.09* (0.04–0.15)
MA7 Ln (Missed interaction)			−0.01 (− 0.01–0.00)	
Time in the study (days)	−0.00* (− 0.01 - − 0.00)	−0.00 (− 0.00–0.00)	−0.00 (− 0.00–0.00)	-0.00* (− 0.00 - -0.00)
Time^2^ (days^2^)	0.00* (0.00–0.00)	0.00 (− 0.00–0.00)	0.00* (0.00–0.00)	0.00* (0.00–0.00)
Ln (reaction)	0.01* (0.00–0.03)	0.01 (− 0.00–0.03)	0.00 (− 0.00–0.00)	0.01* (0.00–0.02)
Workdays (vs. weekends)			0.01* (0.00–0.02)	0.02 (− 0.03–0.07)
Season (winter ref.)				
Spring	− 0.39* (− 0.59 - -0.19)	−0.36* (− 0.57 - -0.16)	0.06* (0.02–0.09)	−0.12* (− 0.24–0.01)
Summer	−0.15 (− 0.40–0.10)	−0.22 (− 0.47–0.05)	0.05* (− 0.48–0.04)	−0.00 (0.00–0.10)
Fall	−0.61* (− 0.94 - -0.29)	−0.26	− 0.19*	−0.24*
		(−0.60–0.07)	(−0.26 - -0.13)	(− 0.44 - -0.03)
Region (SE ref.)				
NE	−1.34 (−2.83–0.15)	−0.60	0.08	−0.42
		(−2.03–0.82)	(− 0.29–0.45)	(−1.49–0.64)
M	−0.57 (− 1.61–0.48)	0.08	0.06	0.04
		(−0.91–1.07)	(−0.19–0.32)	(−0.70–0.79)
West	−0.54 (− 1.73–0.65)	−0.64	0.10	−0.10
		(−1.76–0.48)	(− 0.20–0.40)	(−0.94–0.74)
Season#Region (South ref.)				
Spring#NE	0.59* (0.25–0.92)	0.89* (0.55–1.24)	0.04 (−0.02–0.10)	0.33* (0.12–0.54)
Spring#M	0.31* (0.07–0.56)	0.26* (0.01–0.51)	0.01 (−0.03–0.06)	0.09 (− 0.07–0.24)
Spring#W	0.36* (0.10 -– 0.63)	0.34* (0.07 -– 0.61)	−0.12* (− 0.17 - -0.07)	−0.00 (− 0.17 -– 0.16)
Summer#NE	0.68* (0.26–1.09)	0.78* (0.35–1.20)	−0.00 (− 0.08–0.08)	0.31* (0.05–0.56)
Summer#M	0.37* (0.05–0.69)	0.26 (−0.06–0.59)	0.04 (− 0.02–0.10)	0.13 (−0.07–0.33)
Summer#W	0.36* (0.02–0.70)	0.22 (− 0.13–0.56)	− 0.09* (− 0.15 - -0.02)	−0.03 (− 0.24–0.18)
Fall#NE	0.77* (0.16–1.38)	0.41 (− 0.21–1.04)	0.23* (0.12–0.35)	0.28 (−0.10–0.65)
Fall#M	0.82* (0.40–1.24)	0.49* (0.06–0.92)	0.24* (0.16–0.32)	0.27* (0.01–0.53)
Fall#W	0.71* (0.31–1.11)	0.56* (0.15–0.97)	0.11* (0.04–0.19)	0.25* (0.01–0.50)
RA	− 0.79* (− 1.73–0.15)	−0.68 (− 1.58–0.21)		−0.44 (−1.11–0.24)
Fib	3.03* (1.07–4.99)	2.50* (0.69–4.31)	0.42 (− 0.03–0.88)	2.19* (0.81–3.56)
Constant	7.74* (3.47–12.00)	4.81* (3.61–6.00)	1.49* (0.93–2.05)	3.72* (2.73–4.71)
Observations	5806	5806	5806	5806
Number of participants	121	121	121	121

95% confidence intervals in parentheses

*p < 0.05

*HAQ-II* Health Assessment Questionnaire II; *PAS-II* Physical Activity Scale II; *PRO* Patient reported outcome; *RD* Rheumatic disease; *USD* United States Dollars; *MA7 Ln* Moving average over 7 days of the natural log of (variable); *Ln* Natural log; *NE* Northeast; *M* Midwest; *W* West; *RA* Rheumatoid Arthritis; *Fib* Fibromyalgia

## Discussion

This study included 446 individuals with RMDs participating in a study to measure passive sensing data and PROs using smartphones. With this information, we sought to compare user phone activity (time of use, number and duration of calls, number, and length of text messages) and smartphone sensing activity (mobility, distance travelled, geographic location) in individuals with RMDs (RA and non-RA), and assess associations between these passively collected behavioral data and participants PRO disease severity (PAS-II, pain, and HAQ-II).

Longer reaction times, interaction diversity, and higher mobility were associated with worse PRO disease severity. It is unsurprising that slower reaction times would be consistent with more pain and disability. Previous research on other chronic and disabling conditions has associated slower reaction time, as measured through smartphone sensing data, as predictive of disease severity [17] It is unclear why more mobility and miles traveled, and more interaction diversity would be associated with worse PRO disease severity. We would expect that individuals with more severe and disabling disease would be less mobile with less interaction, due to limited range of motion and pain. One possibility is that those with more symptoms may need to seek more care, and this would require interacting with more phone numbers and traveling greater distances. Alternatively, perhaps a certain level of mobility and interaction contributes to an overexerted state that contributes to increased RMD symptomatology. Associations between reaction time, interaction diversity and mobility with worse PRO were statistically significant, but it is unclear if the differences demonstrate clinical significance.

Longer length of SMS text message was associated with better PRO outcomes (less pain, better function, less disease activity). Similar results have been found in depressed individuals during symptom remission, indicating individuals may be better able to formulate ideas, concentrate, and have the motivation to compose and respond to text messages when feeing well [18]. Although this finding was statistically significant, the magnitude of linear associations was modest.

Participants in this study showed good levels of adherence which holds promise for future interventions using passive behavior measures in self-management and clinical follow-up. Examining predictors of discontinuation of response to smartphone data collection is also important as additional reminders can encourage continued participation.

### Limitations

Limitations include the observational nature of the study and self-selection of the sample. Data collection was completed over 5 years ago, and thus, results were limited by the technology at the time. Participants were active participants in the longitudinal study FORWARD who owned smartphones, limiting the generalizability of these findings. At the time of study, the Android operating system was the only system where we could evaluate all passive variables, therefore limiting the sample further. The analyses also assumed patients were carrying their phone at all times (for mobility). The recruitment strategy included extending participation of individuals in Phase I which reduced the number of unique participants. Additionally, the novel nature of our study required new analysis techniques for both PROs and sensing data, which have not been previously tested.

## Conclusion

A significant challenge with RMDs like RA is the unpredictability of symptom flares. While many studies have been conducted to predict the prodrome for rheumatic disease flares, each person has specific triggers and an individualized course of symptom flare and quiescence. The behavioral patterns associated with the RA prodrome and symptom experience have the potential to be measured and predicted with passively collected smartphone data. Smartphones are a feasible option to collect data about disease activity in individuals with both rheumatic diseases and other chronic health concerns. Correlating PROs with passive measures can strengthen our knowledge about both variables. Future work should focus on ways to passively collect PRO-type data that has clinical relevance in the individual’s treatment plan. Future research may find that smartphone passive data may be a less intrusive means to identify worsening disease burden in participants with rheumatic and other disease states. Additional studies on the behavior of individuals and their rheumatic symptomatology and the measurement of these variables using smartphone technology should confirm and expand these findings.

## Data Availability

The data that support the findings of this study are available upon reasonable request. The data are not publicly available due to their containing information that could compromise the privacy of research participants. For data requests contact Kaleb Michaud, kmichaud@unmc.edu.
